# Evaluating the role of silver nanoparticles on acrosomal reaction and spermatogenic cells in rat

**Published:** 2013-05

**Authors:** Sayyed Mohsen Miresmaeili, Iman Halvaei, Farzaneh Fesahat, Asghar Fallah, Narges Nikonahad, Mohaddeseh Taherinejad

**Affiliations:** 1 *Yazd Academic Center of Education, Culture and Research Higher Education Institute, Yazd, Iran.*; 2 *Department of Biology, Science Faculty, Zahedan, Iran.*; 3 *Research and Clinical Center for Infertility, Shahid Sadoughi University of Medical Sciences, Yazd, Iran.*; 4 *Faculty of Medicine, Shahid Sadoughi University of Medical Sciences, Yazd, Iran.*; 5 *Faculty of medicine, Tehran University of Medical Sciences, Tehran, Iran.*

**Keywords:** *Nanoparticle*, *Spermatozoa*, *Spermatogenesis*, *Acrosome reaction*

## Abstract

**Background: **Nanoparticles have wide range of application while there are some reports regarding their probable effects on male reproductive system and spermatozoa.

**Objective:** The aim of this study was to evaluate the effect of different doses of silver nanoparticles (AgNPs) (70nm) on acrosome of rat spermatozoa and number of spermatogenic cells.

**Materials and Methods: **In this experimental study, in experimental group, 32 male wistar rats (8 rats/group) received oral feeding AgNPs every 12 hr in one spermatogenesis period (48 days) by means of gavages in 25, 50 , 100 and 200 mg/kg concentration (experimental groups 1-4, respectively). The control group (8 rats) was treated on schedule with distilled water. Spermatozoa were stained by triple staining protocol for acrosome reaction. Histological evaluation on testis sections was performed using tissue processing and hematoxylin-eosin (H&E) staining.

**Results:** There was significant difference between the control group and the experimental group 1 for acrosome reaction (11.00±0.00 and 24.25±3.68, respectively, p=0.01). There was only significant reduction in spermatogonia cells in experimental group 4. Experimental groups 2, 3 and 4 showed a significant reduction in the number of primary spermatocytes and spermatids as well as spermatozoa. But there were no significant differences between different groups for Sertoli cell number and seminiferous tubule diameter.

**Conclusion:** It seems that Ag NPs have acute and significant effects on spermatogenesis and number of spermatogenic cells and also on acrosome reaction in sperm cells. More experimental investigations are necessary to elucidate better conclusion regarding the safety of nanoparticles on male reproduction system.

## Introduction

Nanotechnology is the science in which materials are created and manipulated in nanoscale levels (1-100nm). These nanoscale products have unique properties. For example, they have ultra-small size, large surface area to mass ratio, and high reactivity. Because of these properties, they are used as therapeutic and diagnostic agents, drug delivery systems, medical devices, food products and cosmetics. Increasingly, metal nanoparticles represent their benefits in both conventional technology and biomedical industries (1, 2). Also, the nanoparticles are small enough to penetrate even to a very small capillary in the body (3). They can move across in the body boundaries, penetrate to the cells, accumulate there, and may cause cancer, which inhibits fertility and creates defective offspring (4, 5). 

Silver nanoparticles (Ag NPs) is one of the most popular nanomaterials have been used in material science, such as one of the constituent elements of dental alloys, catheters, implant surfaces and for treating of wounds and burns related infections, as well as in drug delivery in cancer and retinal therapies (6, 7). But, there are some concerns about the safety of using Ag NPs in biomedical and in other industries (8, 9). One concern is about the probable impacts of Ag NPs on remote organs. The toxicity of Ag NPs was evaluated by in vivo studies which are shown that these materials may redistribute in different organs and have systemic complications such as weight loss and inflammation (10, 11). Theoretically, Ag NPs may have some negative effect on human health and environment and their probable impact(s) on the male reproductive functions is remained to be clarified. 

There are rare studies regarding the effects of Ag NPs on testis as well as sperm function. In a recent study, the acute effects of intravenously administered a single bolus dose of Ag NPs showed on rat spermatogenesis and seminiferous tubules morphology (12). Another investigation was in vitro evaluating of buffalo sperm parameters. The experiments revealed a dose-dependent decrease in sperm viability with no change in sperm motility suggesting the Ag NPs up to 50 μg/ml concentration can be used for biological applications (13). To our knowledge there is no accurate study about the effect of Ag NPs on sperm acrosome reaction. 

Our main goal was to evaluate the impact of different doses of Ag NPs on sperm acrosome status as well as the number of spermatogenic cells (spermatogonia, primary spermatocytes, spermatid, spermatozoa, and Sertoli cells) following oral administration in rats.

## Materials and methods

**Materials**

**Figure 1:**
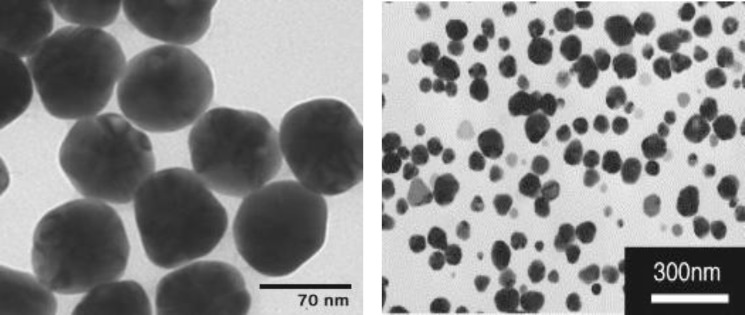
Scanning electron microscopy of Ag NPs. Materials with size of less than 100 nano meter (nm) are considered as nanoparticles. As the both figures verify, the materials used in this study were categorized to nanoparticles

The Ag NPs (Ag- 70 nm) were provided by Research Institute of Payamnour Yazd University (Figure 1). At the first, Ag NPs were dissolved in Phosphate Buffered Saline (PBS) and then used in final concentration of 25, 50, 100 and 200 mg/kg.

**Animals**

In this experimental study, 32 male Wistar rats weighting between 200-250g at the age of 45-50 days were used. Acrosome reaction was evaluated in 3 experimental groups (Exp. 1, 2 and 3). For this purpose, the animals were assigned to 1 control and 3 experimental groups (8 rats / group). The animals were cared for in accordance with the guideline of laboratory animals at our university and the experimental proposal was approved by our university ethics committee. The experimental groups were received oral feeding of Ag NPs every 12hr in one spermatogenesis period (48 days) by gavage in 25, 50, 100 and 200 mg/kg concentration. The control group was treated on schedule with distilled water. Experimental and control groups were kept under standard conditions (12-hour light/dark cycle at 22-24^o^C, with free access to water and food).

**Epididymis sampling and histology evaluation**

After anesthetizing the rats by ketamine/xylazine (60 mg/kg and 6 mg/kg, respectively, Rotexmedica GmbH, Germany), their abdominal area was sterilized by 70% ethanol. The tail of epididymis was separated and washed with Ham’s-F10. Then, it was dissected in the same media and incubated for 30 min at 37^o^C. For evaluating and counting the spermatogenic cells (spermatogonia, primary spermatocytes, spermatid, spermatozoa, and Sertoli cells) perfusion and fixation procedure were performed for tissue samples. 

The samples were fixed by transcardial perfusion with 200 mL of saline (pH, 7.2), followed by 400 mL of 4% paraformaldehyde and then cut into blocks and embedded in paraffin. A series of 4µm thick sections at various levels (100-µm intervals) was cut from each block. After staining by hematoxylin–eosin (H&E), tissue sections were examined by light microscope (Olympus, Tokyo, Japan) at a magnification of 100×. 

All types of cells were counted separately for control and experimental groups from 10 seminiferous tubules that were randomly selected. Only round tubes were counted and the tubes with either oval or elliptical and lost cells were not considered. Different sections from the seminiferous tubules were prepared in order to determinate the seminiferous tubules changes and spermatogenic cells status (morphology, number and adhesion). Histological evaluation was approved by an expert histologist. 

**Acrosome triple staining**

Acrosome triple staining was done according to previously described method with some modifications (14). Spermatozoa suspension was diluted with an equal volume of 2% trypan blue (Sigma Aldrich Chemie, Steinheim, Germany) and incubated at 37^0^ C for 15 min. Suspension was centrifuged (600 g for 5 min) at room temperature (RT). After several washings, the pellet was resuspended in 1 mL PBS (PH=7.4) and centrifuged again as same as before. 

Sperm fixation was done with adding 2mL of 3% glutaraldehyde (Darmstadt, Germany) in 0.1 M cacodylate (Merck, Germany) buffer for 30-60 min. After fixative removing (centrifuging of suspension at 600 g for 5 min at RT), two smears from each samples were prepared. For staining, the slides were stained with 0.08% bismark brown solution (PH=1.8) and 0.8% rose bengal solution solute in 0.1 M tris buffer (Sigma Aldrich, Germany) (PH=5.3). The slides were dehydrated in ethanol, cleared in xylene, and mounted with entelan. Then, the total 200 sperm were counted for evaluation of acrosome reaction. Four groups of sperm were observed; A: Dead sperm without acrosome reaction= nucleus of sperm was dark-blue and acrosome area was pink. B: Dead sperm with reacted acrosome= nucleus of sperm was dark-blue and acrosome area was light-blue or colorless. C: Alive sperm without acrosome reaction= nucleus of sperm was brown and acrosome area was pink. D: Alive sperm with acrosome reaction= nucleus of sperm is brown and acrosome area is light-blue or colorless.

**Statistical analysis**

SPSS 16 (Chicago, USA) was used for statistical analysis. The data were reported as meanSD. The Kruskal-Wallis test was applied in order to compare between different groups. All the tests were two tails. Statistical significance was accepted at the level of p<0.05.

## Results

**Acrosome reaction assay**

All sperm cells in each slide were counted and classified in A, B, C, and D (64 slides were evaluated). Significant trends only observed for acrosome reaction for rate of grade C in exp.1 compared to control 

(24.25±3.68 and 11.00±0.00, respectively, p<0.01). There was no significant difference between dead sperm cells with or without acrosome reaction in experimental groups compared to control group, while the results were positive for viable sperm cells in acrosome reaction between all control and experimental groups. Also, a dose-dependent increase in Ag NPs was observed only between the 2 and 3 experimental groups in alive spermatozoa with acrosome reaction group (D) (p=0.01, Table I). 

These finding indicated both live sperm with or without acrosom reaction were vulnerable to AgNPs compared to control group. On the other hand, increasing the number of dead sperm with intact acrosome and no acrosome after treatment with AgNPs precisely were consistent with the reduced number of spermatozoa and reflects the fact that AgNPs can impair both sperm cell viability and acrosome reaction.

**The effect of concentration of silver nanoparticle on spermatogenic cells**

**Figure 2:**
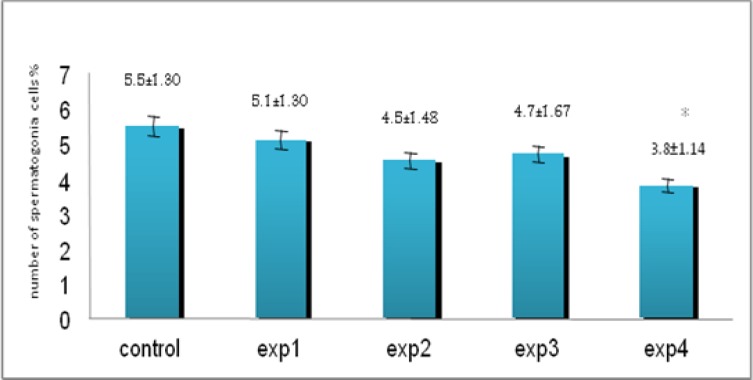
The comparison between control and experimental groups at mean percentage of spermatogonia cells. There was a decreasing trend in experimental groups for spermatogonia cells from control to exp4 (except exp3) and exp4 showed significant difference compared to other groups. _* _Significant reduction has been observed in experimental group 4. (p=0.027) compared with control group

**Figure 3:**
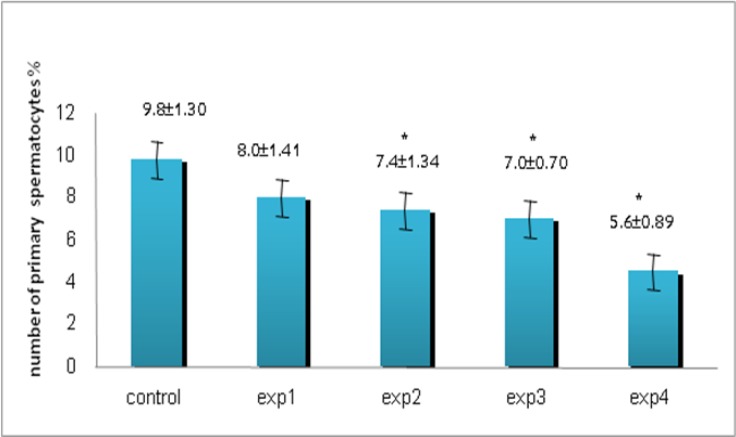
The comparison between control and experimental groups at mean percentage of primary spermatocytes. A decreasing trend was seen from control to exp4 group and exp2, exp3 and exp4 showed significant difference. _* _Significant reduction has been observed in experimental group 2, 3, 4. (p=0.012) compared with control group

**Figure 4:**
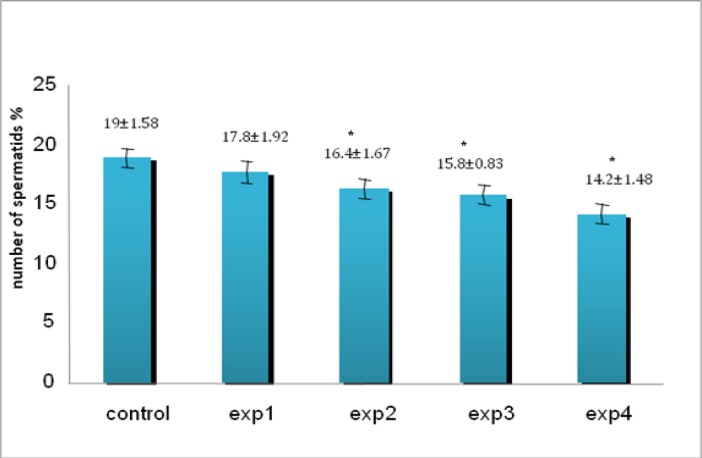
The comparison between control and experimental groups at mean percentage of spermatids. The spermatid cells showed decreasing reduction from control group to exp4 group. _* _Significant reduction has been observed in experimental group 2, 3, 4. (p=0.03) compared with control group

**Figure 5:**
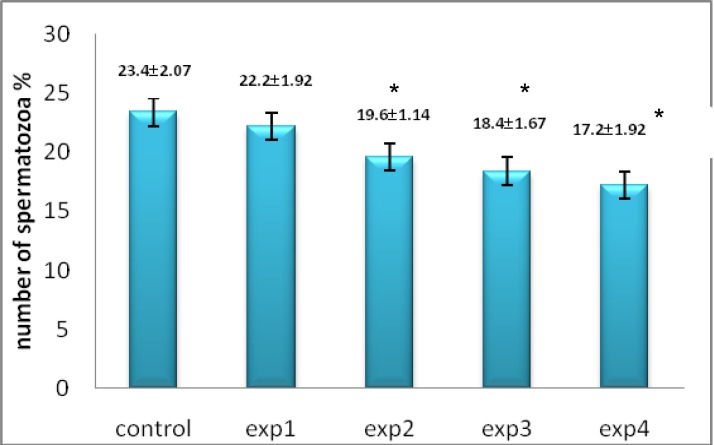
The comparison between control and experimental groups at mean percentage of spermatozoa. Spermatozoa showed decreasing trend in experimental group with increasing dose of silver nanoparticles. _* _Significant reduction has been observed in experimental group 2, 3, 4. (p=0.03) compared with control group

Despite reduction in spermatogonia percentage compared with other groups, it was only seen a significant reduction in experimental group 4 (200 mg/kg) (p=0.027, Figure 2). Microscopic studies showed a significant reduction in number of primary spermatocytes in all experimental groups except experimental group 1 (p=0.012, Figure 3) as well as spermatids (p=0.03, Figure 4) and spermatozoa (p=0.03, Figure 5) compared to control group. 

But there were no significant differences between groups for Sertoli cell number when compared with control group (data not shown).

**Histological assessment**

**Figure 6:**
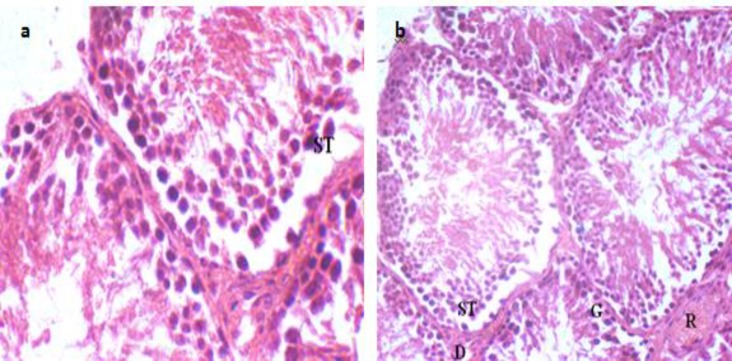
The effect of Ag NPs on seminiferous tubules. a) The separation of primary spermatocytes and spermatogonia cells from tubules wall in experimental group 3 has been observed (magnification 1000 ×).b) The separation of spermatogonia cells and spermatocytes and releasing of sperm precursor cells to mid-duct of seminiferous tubules in experimental group 4 has been shown (magnification 400 ×). R; blood vessel, G; spermatogonia cell, ST ; primary spermatocyte, D ;Leydig cell

In cross-section of testes, a fairly large number of seminiferous tubules have been observed that were surrounded by connective tissue. Despite a little reduction in the seminiferous tubules diameter, there is no significant changes in the diameter in the animals treated with Ag NPs in different doses 48 days after oral administration (the time period of spermatogenesis) (data not shown). But due to releasing the spermatogonia cells, spermatid and primary spermatocytes into the duct of some seminiferous tubules and their separation from the wall were observed in experimental group 3 (Figure 6a) and 4 (Figure 6b) compared with control group, clearly.

**Table I:** Effect of different concentrations of Ag NPs on the percentage of alive and dead spermatozoa (mean ± SD) with and without acrosome reaction in vivo

**Parameters **	**Groups**	**Mean ± SD**	**p-value**
Dead sperm without acrosome reaction (A)			0.496
	Control	19.00 ± 1.41	
	Experimental 1	29.25 ± 15.54	
	Experimental 2	33.66 ± 8.12	
	Experimental 3	31.17 ± 16.80	
Dead sperm with acrosome reaction (B)			0.316
	Control	35.50 ± 0.71	
	Experimental 1	26.75 ± 14.10	
	Experimental 2	39.43 ± 6.53	
	Experimental 3	39.50 ± 14.67	
Alive sperm without acrosome reaction (C)			0.026
	Control^ *^	11.00 ± 0.00	
	Experimental 1^ *^	24.25 ± 3.68	
	Experimental 2	16.66 ± 4.41	
	Experimental 3	15.00 ± 4.00	
Alive sperm with acrosome reaction (D)			0.011
	Control^†^	11.00 ± 0.00	
	Experimental 1^$^	24.25 ± 3.68	
	Experimental 2^ †$^	16.66 ± 4.41	
	Experimental 3	15.00 ± 4.00	

^*^,^ $^,†: p<0.05. Kruskal-Wallis test was applied in order to compare between different groups

## Discussion

In our study, the acute effects of oral administered AgNPs (70nm) at different doses (25, 50,100,200 mg/kg) on the spermatogenic cells count and acrosome reaction in sperm cells on the male Wistar rats were evaluated. Our results showed that oral administration of Ag NPs can impair acrosome reaction in rats. 

The studies were carried out on C18-4 cell line by Braydich-Stoll *et al* showed that nanoparticles such as silver and aluminum nanoparticles were able to cross sperm membrane and connected to mitochondria and acrosome of sperm cells (15). The study results showed that percentage of dead sperm with and without acrosome reaction compared with viable sperm in the same condition and between experimental groups has increased. This increase was dose dependent and sperm with abnormal morphology has reached to maximum at concentration 200 mg/kg. (Table I). It can be attributed to the effect of AgNPs on DNA. It could react with cellular DNA and stimulated inflammation and oxidative damage and cellular dysfunction that created genetic mutation and sperm cells with abnormal morphology (16).

Despite a few reductions in the seminiferous tubules diameter, there are no significant changes in the diameter in the animals treated with Ag NPs in different doses after 48 days (the time period of spermatogenesis in rats) but releasing the spermatid and spermatocytes into the duct of some seminiferous tubules were observed (Figure 6). Many in vivo studies have shown that chemicals and metals, such as chromium, cadmium or lead can decrease the diameter of seminiferous tubules epithelial cells and in consequence the seminiferous tubules lumen (17, 18). 

Our histological evaluation on testes tissues indicated some damaged tubules in all experimental groups that was in line with study by Takeda and Suzuki (19). Also, significant decrease in mean number of primary spermatocytes, spermatids and sperm cells in all experimental groups except group 1 (25mg/kg) may be related with Ag NPs inhibitory role in cell proliferation. Some evidences about the effect of nanoparticles on spermatogonial stem cells (SSCs) indicated that they can cause the reduction of cell proliferation in these cells (20). 

It seems that reduction of FYN kinase (the member of the Src family kinase involved in the proliferation of spermatogonia, which are abundant in Sertoli cells) activity is the main reason of decreasing the cell numbers in spermatogenesis. FYN kinases play a role in the adhesion of spermatogenic cells such as spermatids to Sertoli cells and decreasing these proteins disturbed spermatid adhesion to Sertoli cells and sperm reduction (21).

Our data showed a significant reduction of sperm cell number in all experimental groups except exp. group 1. It might be due to the effect of nanoparticles on cell cycles and significant decrease of sperm precursor cells or release of them to the mid duct of seminiferous tubules. We did not find any significant difference for the number of Sertoli cells in any exp. groups. It may be possible that this slight decrease is related to the increased production of nitric oxide (NO) in Sertoli cells (22). 

In line with our findings, it was shown that TiO2 (Titanium dioxide) nanoparticles could cross the blood-testes barrier (BTB) and form aggregates/agglomerates in Sertoli cells. This in turn, caused a reduction in their number and led to damage and disorganization of the seminiferous tubules. Damaged tubules were observed in scattered seed clusters throughout the testicular tissues (23).

## Conclusion

In conclusion, our study showed the Ag NPs even in small size have acute and significant effects on spermatogenesis and number of spermatogenic cells and also on acrosome reaction in sperm cells. Also, high doses of Ag NPs (100 and 200 mg/kg) had a negative effect on spermatogenesis process and can influence reproductive potential in animal models. More experimental investigations are necessary to elucidate better conclusion regarding the safety of nanoparticles on reproduction system while it seems these materials should be used in medicine and industries with more caution. 
